# Patient and health service delay in pulmonary tuberculosis patients attending a referral hospital: a cross-sectional study

**DOI:** 10.1186/1471-2458-5-122

**Published:** 2005-11-24

**Authors:** Mpungu S Kiwuwa, Karamagi Charles, Mayanja Kizza Harriet

**Affiliations:** 1 Clinical Epidemiology Unit, Makerere University, Faculty of Medicine, P.O Box 7072, Kampala, Uganda; 2 Department of Medicine, Makerere University, Faculty of Medicine, P.O Box 7072, Kampala, Uganda

**Keywords:** pulmonary tuberculosis, health-seeking behavior, treatment delay

## Abstract

**Background:**

Delays in diagnosis and initiation of effective treatment increase morbidity and mortality from tuberculosis as well as the risk of transmission in the community. The aim of this study was to determine the time taken for patients later confirmed as having TB to present with symptoms to the first health provider (patient delay) and the time taken between the first health care visit and initiation of tuberculosis treatment (health service delay). Factors relating to these 'delays' were analyzed.

**Methods:**

A cross-sectional survey, of 231 newly diagnosed smear-positive tuberculosis patients was conducted in Mulago National referral Hospital Kampala, from January to May 2002. Socio-demographic, lifestyle and health seeking factors were evaluated for their association with patient delay (>2 weeks) and health service delay (>4 weeks), using odds ratios with 95% confidence intervals (CI) including multivariate logistic regression.

**Results:**

The median total delay to treatment initiation was 12 weeks. Patients often presented to drug shops or pharmacies (39.4%) and private clinics (36.8%) more commonly than government health units (14%) as initial contacts. Several independent predictors of 'patient delay' were identified: being hospitalized (odds ratio [0R] = 0.32; 95% CI: 0.12–0.80), daily alcohol consumption (OR = 3.7; CI: 1.57–9.76), subsistence farming (OR = 4.70; CI: 1.67–13.22), and perception of smoking as a cause of TB (OR = 5.54; CI: 2.26–13.58). Independent predictors of 'health service delay' were: >2 health seeking encounters per month (OR = 2.74; CI: 1.10–6.83), and medical expenditure on TB related symptoms >29 US dollars (OR = 3.88; CI: 1.19–12.62). Perceived TB stigma and education status was not associated with either form of delay.

**Conclusion:**

Delay in diagnosis of TB is prolonged at the referral centre with a significant proportion of Health service delay. More specific and effective health education of the general public on tuberculosis and seeking of appropriate medical consultation is likely to improve case detection. Certain specific groups require further attention. Alcoholics and subsistence farmers should be targeted to improve accessibility to TB treatment. Continuing medical education about TB management procedures for health providers and improvement in the capacity of TB control services should be undertaken.

## Background

Uganda is one of the global TB high burden countries ranked 20^th ^in the world with an estimated incidence rate of tuberculosis in 2001 of 324 cases per 100,000 and a notification rate of 171 per 100,000 of the population. The estimated proportion of tuberculosis cases that were HIV positive was 35% in 2001. The number of reported TB cases is increasing with 19,016 cases registered in 1991 and 41,831 in 2001[1]. This increase is possibly due to population growth, improvement in case finding or a true increase, as sentinel sero-prevalence surveys in Ugandan hospitals had shown that 45% to 65% of newly diagnosed TB patients were HIV positive[2]. The detection of new sputum smear positive cases was 52%, which is far below the (WHO) case detection target of 70%[3].

In Mulago National Referral TB treatment center located in Kampala, the capital city of Uganda, clinic records show that the majority of tuberculosis patients have had symptoms for two or more months before seeking TB treatment. Delays in diagnosis and initiation of effective treatment increase morbidity and mortality from tuberculosis as well as the risk of transmission in the community. The stigma attached to TB disease may hinder access to timely health care[4].

In patients with TB, the low detection and cure rates may be attributed to limited information about factors contributing to delayed diagnosis in Uganda. Recent work in Mukono [5], a mainly rural district revealed that there is considerable delay in diagnosis of TB. However factors contributing to delay in diagnosis and treatment are likely to vary depending on the populations in their local settings. The aim of this study was to determine the time interval between symptom onset and initiation of TB treatment[6,7] and analyze the determinants of this interval. From this study, it was determined which factors serve as barriers or facilitators for seeking treatment early, how these factors are interrelated and which ones are liable to behavioral change or social encouragement by health education and health promotion.

## Methods

### Ethical considerations

Institutional consent was obtained from the Faculty Research and Ethics Committee of the Medical School (institutional ethical board for the medical school).

### Study setting

The study was carried out at the National Tuberculosis and Leprosy control program (NTLP) center of Mulago hospital situated in the city centre. This treatment center is the principal facility providing in-patient and outpatient TB care in Kampala district. The TB center which is one of Mulago hospital's various departments has an inpatient bed capacity of about 100 beds. The hospital provides services to Kampala's population of about 1,208,544 (Uganda population census 2001) and surrounding districts plus upcountry referrals. TB patients are usually referred to the center from other hospitals, medical centers and private practitioners in and outside Kampala. Most health care units are strategically located within the city, and the transport system has good coverage. The TB control program initiated decentralization of TB management services to the district and sub-district levels, in 1997. The total number of new TB patients including children registered at the center in 2001 was about 4000.

### Study participant selection

We performed a cross-sectional study during a four-month period from January to May 2002 during which a total of 1,382 TB patients were registered at the treatment center. Eligible participants were patients presenting to the center aged 18 years or older, with newly diagnosed sputum smear positive pulmonary tuberculosis. Consenting eligible patients were consecutively enrolled and interviewed by one of three trained research assistants including the principal author, within two days of the pulmonary tuberculosis diagnosis, using a semi-structured questionnaire.

For hospitalized patients, the TB register at the treatment centre was used to identify eligible patients. Such patients were then located on the ward for consent to participate in the study. Most interviews were conducted in the main local language of the area (Luganda), or in English. Re-treatment patients and those with sputum negative disease were excluded from the study. The questionnaire was pre-tested among TB patients prior to the start of study, with modifications incorporated in the final version. The questionnaire collected information on the following attributes including: (a) Demographic and socio-economic variables: age, sex, occupation, education level, and family size; (b) Physical factors: symptoms and their duration, sputum results and chest radiographic findings); (c) Psychological factors: patient beliefs, perceptions, attitudes and knowledge about tuberculosis; (d) Institutional or health service factors: distance to health facilities, costs of travel and medical expenditure on treatment of TB related symptoms. Each interview schedule lasted approximately 40 minutes per participant and allowed for careful probing of responses to minimize recall bias. Other data sources were case notes and referral letters. A chest physician interpreted chest radiographic findings and regular supervision of the interviewers was conducted throughout the study period.

Participants were asked to estimate the time in months or weeks they had been experiencing the major presenting symptoms – evening fevers, night sweats, chest pain, difficulty in breathing, weight loss, loss of appetite or generalized weakness along with the cough episode leading to initiation of tuberculosis treatment (total delay). Patient delay was the estimated time interval between symptom onset and the first medical consultation and the time taken from then until the diagnosis was confirmed and treatment started was considered as health service delay[6,7]. In our study a health provider was defined as any person consulted by the patient about his/her sickness who prescribed any form of medication. These included dispensers, pharmacists, medical staff and herbalists or traditional healers. The estimated sample size of 231 patients was obtained assuming, from prior local studies that 80% would have a total delay of more than 4 weeks [8]. A total delay of more than four weeks was considered, as national guidelines suggest that patients coughing for more than 3 weeks should be investigated further. Limitations of our study include imprecise estimates of delay due to recall bias or in some cases, lack of case notes and referral letters to verify the accuracy of information given, and the single center studied.

### Data management and analysis

In order to ensure data quality, the data was double entered into a computer, and the two copies of the data verified. Delay in weeks was presented as medians and proportions. Patient delay (classified as presentation after 2 weeks of the onset of symptoms) and health service delay (when patient was delayed >4 weeks between initial contact with health provider and treatment onset) were compared for different sub-groups using odds ratios and 95% CI. To identify factors independently associated with patient delay and health service delay, a multivariate logistic regression analysis with delay time dichotomized as described above was performed. When appropriate, non-parametric tests (Chi-square, Mann-Whitney and Kruskal-Wallis tests) were used where assumptions of normality were unmet. Statistical significance was taken as P < 0.05.

## Results

### Socio-demographic characteristics of the participants

Two hundred thirty one eligible adult newly diagnosed smear positive TB patients were recruited into the study. The mean age was 30.7{Standard deviation (SD) = 9.4}, with a median age of 30 years. The mean age of the males 32.2 (SD = 9.2) years and females 28.7 (SD = 9.2) years were significantly different (p < 0.05). Seventy-two (25.5%), were hospitalized at the time of diagnosis. The mean family size was 4(SD = 2.4) with a range of (1–11). One hundred and thirty-nine had at most post-primary education regardless of gender. Occupation wise 28 (12.1%) were subsistence farmers, 56 (24.2%) skilled workers and 115 (49.8%) unskilled workers including traders and housewives. Of those who did not earn any income, 15 (11.4%) were more likely to be male compared to 36 (36.4%) of the females (P < 0.001). The average medical expenditure on treatment of TB related symptoms was 30 US dollars range (0–412) (Table 1).

**Table 1:** Socio-demographic characteristics and distribution of the various time delays among the participants*

**Characteristic**		**Patients, No. (%)**	
Age, mean (SD), y		30.7 (9.4)	
Sex			
Male		132 (57.1)	
Female		99 (42.9)	
Hospital admission status			
Hospitalised		72 (25.5)	
Outpatients		159 (74.5)	
Highest education level attained			
None		21 (9.1)	
Primary school		118 (51.1)	
Secondary		58 (25.1)	
Post-secondary		13 (5.6)	
Tertiary		21 (9.1)	
No. of persons in Household			
(1)		24 (10.4)	
(>1)		207 (89.6)	
Weekly Alcohol consumption			
Daily		35 (15.2)	
Sometimes		47 (20.3)	
Rarely		26 (11.3)	
Never		123 (53.2)	
Main Occupation			
Subsistence farming		28 (12.1)	
Unskilled workers		115 (49.8)	
Skilled workers		56 (24.2)	
Students		20 (8.7)	
Unemployed		12 (5.2)	
Medical expenditure on TB related symptoms, US $			
0–29		123 (53.2)	
>29		108 (46.8)	
Distance from home to treatment centre, km			
<25		197 (85.3)	
>25		34 (14.7)	

**Distribution of the time delays**			
	Patient delay	Health service delay	Total delay

Median, wk	1.0	9.0	12.0
Interquartile Range, wk	0.9–4.0	5.3–15.3	8.0–20.0
Delay >2 wk	70 (30.3)	207 (89.6)	231 (100)
Delay >4 wk	40 (14.7)	188 (81.4)	210 (90.9)

*Values are number % unless otherwise indicated

### Clinical characteristics of the participants at presentation

The mean performance status (Karnofsky score) which is a measure of the degree of debility, of the respondents was 80 (SD = 10). Of our participants, symptoms experienced included; hemoptysis 63 (27.3%), fever 78.4%, chest pain 68.4%, anorexia, 57.6%, exertional dyspnoea 74%, fatigue 77.9%, and cervical lymphadenopathy 6.5%.

Fourteen (25%) of the hospitalised patients had ≤ 3 affected lung zones compared to 22 (13%) of the out-patients (OR = 2.20; CI: 1.05–4.72). Participants who ever had hemoptysis before diagnosis had a longer delay to initiation of TB treatment (Mann-Whitney test P-value = 0.019). Disease severity as measured by AFB (Alcohol Acid Fast Bacilli) sputum smear grades, was also associated with the duration of delay to treatment. (Kruskall-Walis test P < 0.001). It was also observed that presence of cavitary disease was associated with prolonged delay to initiation of treatment (Man-Whitney test P < 0.001).

HIV sero-status information assessed by self-report was only available in 56 (24%) of the respondents, in which an association with delay could not be established. However self reported HIV positive sero-status results were more common among hospitalised participants 18 (90%) compared to 10 (27.8%) in the out-patients (OR = 23; CI: 4.57–119.77).

Self reported HIV positive patients were more likely to be female 20 (69.0%) vs. male 8 (29.6%), (OR = 5.30; CI 1.69–16.40); and were un-likely to have cavitary disease 24 (85.7 %) vs. 13 (46.4%) of the self reported HIV sero-negatives. (OR = 6.92; CI: 1.90–25.23).

### Distribution of the various time delays

The time delays experienced by the patients are described in Table 1 as well. Health service delay was the most frequent type of delay observed and the greatest contributor to total delay. The total delay to treatment was more than one month in 210 (90.9%) and exceeded 2 months in 65.8% of the cases.

### Place of first presentation

Table 2 shows the first health facility visited by the participants for their illness and the corresponding time delays. Seventy six percent of the patients had first presented to a private clinic or pharmacy/drug shop. Health service delay was longest (13.7 weeks) for those who first approached government health centres for their sickness. Of those who initially presented to government hospitals for treatment, 46.2% were investigated for TB using sputum microscopy; and 69.2% of them, were unlikely to change to another health facility. The prevalence of self-referral to the NTLP centre was 33% and family members were seen to be an important source of influence in seeking treatment in 53% of the cases.

**Table 2:** First health facility visited by the participants for their illness and corresponding median time delays in weeks

First health facility visited	Number	%	Patient delay	Health service delay	Total delay
Pharmacy/Drug shop	91	39.4	1.0	10.6	12.0
Private clinic	85	36.8	1.0	8.4	12.0
Traditional healer	21	9.1	3.0	9.9	12.0
Government hospital	13	5.6	4.0	7.1	12.0
Health center	10	4.3	3.0	13.7	22.0
NTLP center	9	3.9	10.0	0.0	10.0
Non Governmental hospital*	2	0.9			

*Delay times not stated because cases were very few.

### Respondents' awareness of TB symptoms and perceived causes

The most frequently mentioned TB symptom was persistent cough (40.7%) and wasting (25.1%) and fever (9.5%). One hundred and fifteen (50%) patients were not aware of any possible causes of TB, and only 7.4% mentioned that germs cause TB. As noted, there was a low level of awareness of TB symptoms and it's causes in this study population. About 56% of the respondents acknowledged that TB patients are stigmatised in their communities. For instance, 40% of participants would not want to be near a TB case.

### Factors associated with patient delay

Table 3 presents un-adjusted and adjusted odds ratios for patient delay (>2 weeks) for a number of socio-demographic, lifestyle and health seeking factors. Of all the factors investigated, we found that hospitalized patients had a shorter delay to seeking treatment compared to out-patients (OR = 0.38, 95% CI, 0.18–0.81). Notably subsistence farmers delayed longer seeking help for treatment compared to the rest of the respondents (OR = 4.70; CI: 1.67–13.22); and were more likely not to have had post-primary education 27 (96.4%), compared to the other participants 112 (55.2%), (OR = 21.94; CI: 2.93–164.55). Other independent predictors of patient delay included: daily alcohol consumption (0R = 3.70; CI: 1.57–9.67) and misperceptions that TB is caused by smoking (OR = 5.54; CI: 2.26–13.58). Compared with males, females had a shorter delay seeking help for symptoms, although this finding did not reach statistical significance. Severity of initial symptoms and age had no effect on patient delay.

**Table 3:** Relationship between socio-demographic, lifestyle and health seeking factors to Patient delay. Both unadjusted and adjusted odds ratios are shown (n = 231)

**Variable**	**n**	**% Patient delay >2 weeks**	**Unadjusted odds ratio (95% CI)**	n*	**Adjusted odds ratio† (95% CI)**
Age 18–40, y	205	30.2	0.98 (0.40–2.40)		NA
Male	132	35.6	1.83 (1.02–3.29)		NA
Hospitalised	59	16.9	0.38 (0.18–0.81)	56	0.32 (0.12–0.80)‡
Marital status separated/single	125	30.4	1.01 (0.58–1.77)		NA
Post primary education Level	92	25.0	0.65 (0.36–1.18)		NA
>1 Household persons	207	28.5	0.47 (0.20–1.11)		NA
Daily Alcohol consumption	38	51.5	2.93 (1.41–6.11)	34	3.70 (1.57–9.76)‡
Subsistence farming	28	46.4	2.22 (1.02–5.00)	26	4.70 (1.67–13.22)‡
Perceived smoking as cause of TB	34	55.9	3.63 (1.72–7.66)	34	5.54 (2.26–13.58)‡
>2 Health seeking encounters per month	76	13.2	0.24 (0.12–0.50)	72	0.15 (0.06–0.36)‡

*Number included in forward stepwise logistic regression method.

†Odds ratios for the variables appearing at the final step of forward stepwise selection.

‡P < 0.05; CI indicates confidence interval; NA not applicable.

### Factors associated with health Service Delay

The median health service delay was 9 weeks range (0.1–135.6). Only fifty-eight (36%) of the patients were diagnosed and initiated on treatment within one month following initial contact with a health provider. Of the factors investigated, these were the three independent predictors of health service delay >4 weeks, after adjusting for other variables (Table 4) namely: medical expenditure on treatment of TB related symptoms > 29 US dollars, (OR = 3.88), >2 multiple health seeking encounters per month (OR = 2.74) and residing alone (OR = 0.31). Presence of hemoptysis at onset was associated with health service delay although this aspect did not reach statistical significance.

**Table 4:** Relationship between socio-demographic, lifestyle and health seeking factors to Health service delay. Both unadjusted and adjusted odds ratios are shown (n = 231)

**Variable**	**n**	**% Health service delay >4 weeks**	**Unadjusted odds ratio (95% CI)**	n*	**Adjusted odds ratio† (95% CI)**
Age 18–40, y	205	81.0	0.77 (0.25–2.37)		NA
Male	132	81.1	0.95 (0.49–1.86)		NA
Hospitalised	59	86.4	1.63 (0.71–3.75)		NA
Marital status separated/single	125	82.1	1.09 (0.56–2.12)		NA
Post primary education level	92	83.5	1.40 (0.72–2.73)		NA
Single Household person	24	66.7	0.41 (0.16–1.03)	23	0.31 (0.11–0.84)‡
Daily Alcohol consumption	38	85.7	1.44 (0.53–3.97)		NA
Subsistence farming	28	89.3	2.05 (0.59–7.11)		NA
Perceived smoking as cause of TB	34	87.5	1.62 (0.20–13.56)		NA
>2 Health seeking encounters per month	76	89.5	2.48 (1.09–5.65)	72	2.74 (1.10–6.83)‡
Medical expenditure on TB related symptoms (>29 US $)	60	88.3	2.02 (0.85–4.82)	60	3.88 (1.19–12.62) ‡
Hemoptysis at onset	33	93.9	4.05 (1.02–15.90)		NA

*Number included in forward stepwise logistic regression method.

†Odds ratios for the variables appearing at the final step of forward stepwise selection.

‡P < 0.05; CI indicates confidence interval; NA not applicable.

## Discussion

### Delay in diagnosis and initiation of TB treatment

This study from Kampala Uganda highlights the prolonged delay from onset of patients' symptoms until initiation of TB treatment at Mulago hospital. Health service delay (74%) represented the main component of the overall total delay, reflecting an inadequacy of the clinical services to diagnose TB among symptomatic individuals. The median total delay to treatment of 12 weeks observed in this study is similar to that seen in other studies[6,9,10]. In Uganda [5], Oola (2001) found a median total delay of 17 weeks among both smear positive and smear negative TB patients.

Seventy percent of the respondents reported visiting a health provider within 2 weeks of onset of signs and symptoms. This short patient delay could reflect good awareness of health problems and easy access to health care in the study population. However 21 (9%) of patients sought the initial treatment 2 months after symptom onset. Often misinterpretation of initial symptoms results in actions (patients' reliance on self medication and pharmacy or drug shop attendance) that may delay seeking of appropriate medical care. The greater proportion of health service delay found in our study is similar to prior findings from studies in Ghana, Botswana and Gambia[7,9,10], but contrary to findings from South-Africa and Tanzania[11,12]. This long health service delay conceivably reflects insufficient knowledge of the signs and symptoms of TB among different types of health providers and the general population. The shorter patient delay found in our study, contrasts with findings of Oola (2001), in which patient delay was prolonged[5]. Most of the patients (61.5%) in Oola's study were rural based, compared to the more urban setting of the present study. Rural residence has been found to be a risk factor for late diagnosis because of poorer access to health care and differences in education levels between rural and urban areas[10,11].

### Factors associated with patient delay

In this present study, perception of smoking as a cause of TB was associated with prolonged patient delay. This may be because cough appearing in smokers is often attributed to smoking, resulting in delays seeking help for TB related symptoms. By contrast, in Philippines[13], there was no relationship between perceived causes of TB and health-seeking due to potential recall and selection biases. Education status had no effect on delay to treatment initiation as established elsewhere[10,11]. However subsistence farmers were found to have long patient delays probably due to lack of education and poverty. Similarly, alcoholics had prolonged patient delays. Gender had little impact on delay[14], though other studies have confirmed prolonged delays to initiation of treatment among females[5,7,11]. However larger studies should be undertaken to explore the effect of gender on access to health care. Severity of initial symptoms had no effect on delay possibly due to recall bias and individual variations in perception of disease.

### Factors associated with health service delay

Our study revealed that the private health system is the common first choice of care for TB patients in Kampala district, with approximately 75% of individuals presenting to a private clinic, pharmacy or drug shop initially. In contrast, 75% of the patients in South Africa used the public health system as the initial contact[11]. This may be partly related to the health-care service quality. The public health sector in Uganda offers a free health service in agreement with the government health policy, though existing under-funding of this sector leads to insufficient utilities and consequently discourages it's optimal use. Longer delays to treatment were experienced among patients originating from peri-urban localities in which limited access to primary health care services significantly contributes to late diagnosis[7,10].

Multiple health seeking encounters contributed to the prolonged duration of health service delay along with the associated medical costs. A low level of clinical suspicion of TB by health providers and failure to order proper investigations or refer patients to 'higher level' health units contributes in a major way to health service delay. Patients often need to return to the same providers of healthcare or seek advice from others, because of persistent symptoms[6,15].

### Clinical features associated with delay to treatment

Our study found that patients who required hospitalisation prior to TB diagnosis had shorter delays to treatment and less advanced disease, compared to out-patients. This shorter delay to treatment is partly explained by the finding that hospitalisation was HIV associated. In Ghana [7] hospitalised patients had more advanced disease and prolonged delays to treatment, though HIV status was not documented. A disparity in the HIV epidemic transitions in both countries is a possible explanation for this difference. The presence of limited cavitary disease among known HIV positive patients is known to be related to diminished immune function. Illness severity as measured by chest radiography and sputum smear grades was found to be associated with the duration of delay to treatment. Advanced disease has been found to correlate with mortality and chronic morbidity [7].

## Conclusion

Patient and health service delays contribute significantly to delays in patients accessing treatment. Substantial reduction in case detection delays may be achieved through; 1) More specific and effective health education of the general public on tuberculosis and seeking of appropriate medical consultation; 2) Targeting specific groups of alcoholics and subsistence farmers to improve accessibility to TB treatment 3) Continuing medical education about TB management procedures for health providers; 4) Improvement in the capacity of TB control services. Similar research in the future could serve as a basis for monitoring improvement in the quality of the tuberculosis control program in Uganda.

## Competing interests

The author(s) declare that they have no competing interests.

## Authors' contributions

MSK conceived of the study, wrote the research proposal, conducted the research, did data entry, performed statistical analysis and wrote the manuscript. KC was involved in the write up of the proposal, data analysis and draft of the manuscript. MKH was involved in the write up of the proposal, data analysis and draft of the manuscript.

## Pre-publication history

The pre-publication history for this paper can be accessed here:


